# Supplementary material for: Single-cell network biology characterizes cell type gene regulation for drug repurposing and phenotype prediction in Alzheimer’s disease
Source: PLoS Comput Biol. 2022 Jul 18;18(7):e1010287. doi: 10.1371/journal.pcbi.1010287 (PMC9333448; doi:10.1371/journal.pcbi.1010287)
Supplement: S1 File — Figs A-N. Fig A. (1) Distribution of different edge types within cell type GRNs. (2) An upset plot showing overlaps between the top 10% genes with largest in-degree. The filled dots in the centre matrix indicate the comparison between the respective sets (along the x-axis), and the bars on the top show size of the intersection. Blue and red rows indicate control and AD, respectively. The red arrow at the bottom shows overlaps between both neuronal types across the two phenotypes. (3) Overlaps between central genes in two independent datasets (Mathys et al. and Lake et al.). (4) Heatmaps depicting correlation in gene centrality scores between the full dataset and a reduced dataset consisting 50% of the original samples in the snRNAseq dataset. Fig B. Hierarchy analysis of cell type GRNs. (1) Sankey plots showing overlaps between regulatory hierarchies of cell type control and AD GRNs (Blue: top-level, green: middlelevel, red: bottom-level). Overlaps between (2) top-level, and (3) middle-level TFs across cell types. Fig C. Barplot showing GO BP terms that gain or loss cohesiveness measured as change in network density between control and AD networks of microglia. Fig D. Barplot showing GO BP terms that gain or loss cohesiveness measured as change in network density between control and AD networks of oligodendrocytes. Fig E. Barplot showing GO BP terms that gain or loss cohesiveness measured as change in network density between control and AD networks of inhibitory neurons. Fig F. Barplot showing GO BP terms that gain or loss cohesiveness measured as change in network density between control and AD networks of excitatory neurons. Fig G. Barplots showing the number of co-regulated gene modules (left y-axis) detected at various levels of edge-weight threshold (right y-axis) and minimum module size cut-off (top x-axis) across cell types (x-axis). Fig H. Barplots showing the number of genes included in modules (left y-axis) detected at various levels of edge-weight thresho [file pcbi.1010287.s010.pdf]

Single-cell network biology characterizes cell type gene regulation for drug repurposing and phenotype prediction in Alzheimer's disease

Chirag Gupta[1], Jielin Xu[2], Ting Jin[1,5], Saniya Khullar[1,5], Xiaoyu Liu[5], Sayali Alatkar[1,5], Feixiong Cheng[2,3,4], Daifeng Wang[1,5*]

Supplementary Figures

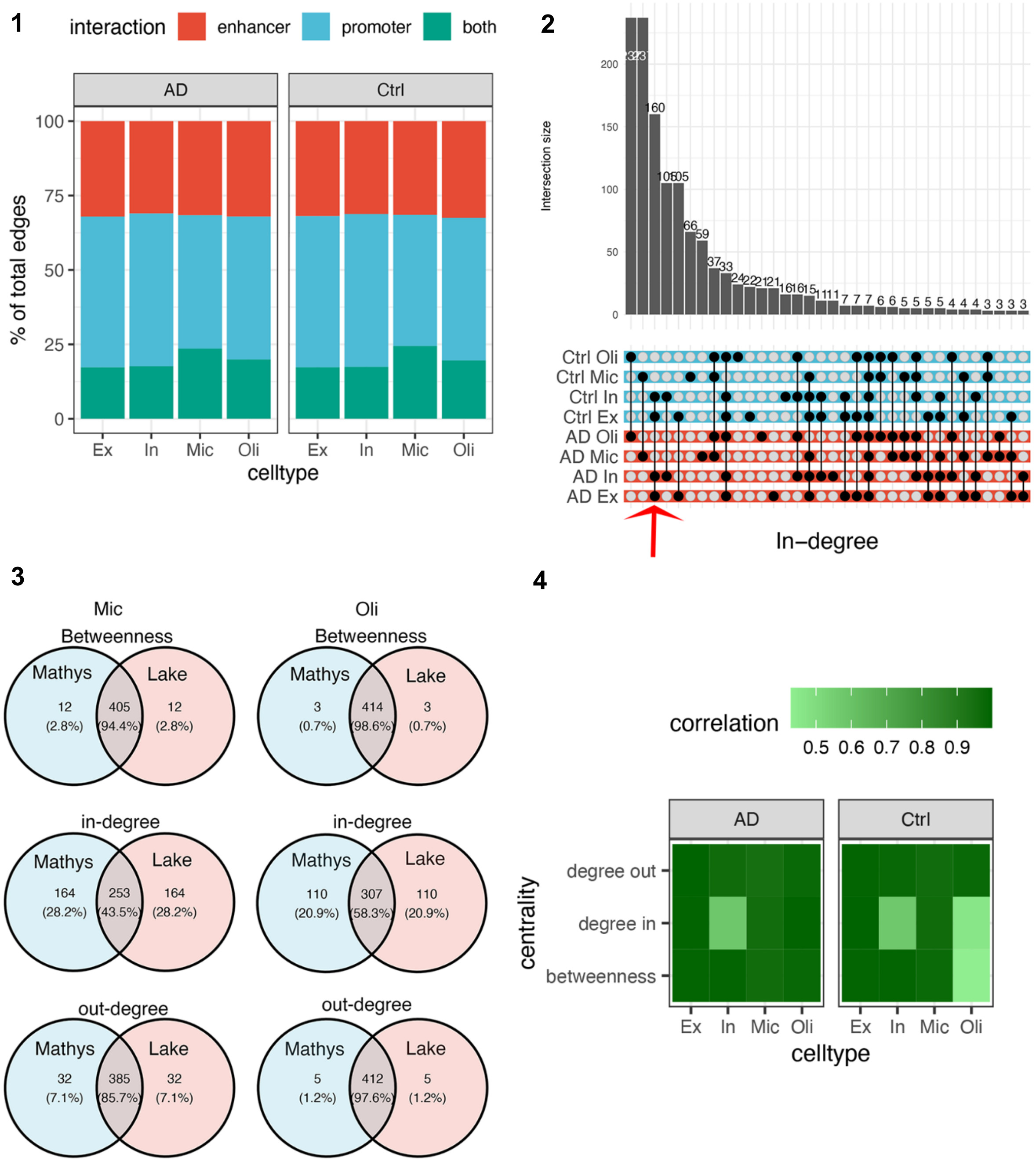

Fig A. (**1**) Distribution of different edge types within cell type GRNs. (**2**) An upset plot showing overlaps between the top 10% genes with largest in-degree. The filled dots in the

centre matrix indicate the comparison between the respective sets (along the x-axis), and the bars on the top show size of the intersection. Blue and red rows indicate control and AD, respectively. The red arrow at the bottom shows overlaps between both neuronal types across the two phenotypes. (**3**) Overlaps between central genes in two independent datasets (Mathys et al. and Lake et al.). (**4**) Heatmaps depicting correlation in gene centrality scores between the full dataset and a reduced dataset consisting 50% of the original samples in the snRNA-seq dataset.

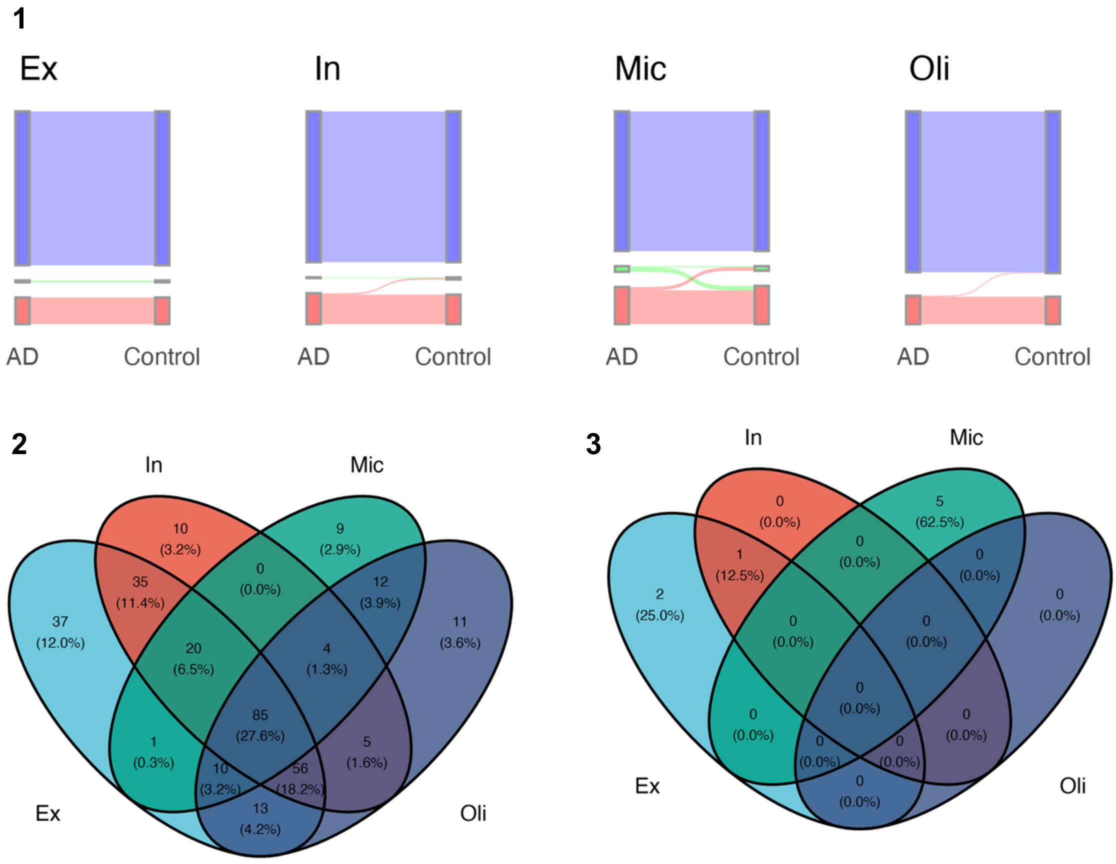

Fig B. **Hierarchy analysis of cell type GRNs.** (**1**) Sankey plots showing overlaps between regulatory hierarchies of cell type control and AD GRNs (Blue: top-level, green: middle-level, red: bottom-level). Overlaps between (**2**) top-level, and (**3**) middle-level TFs across cell types.

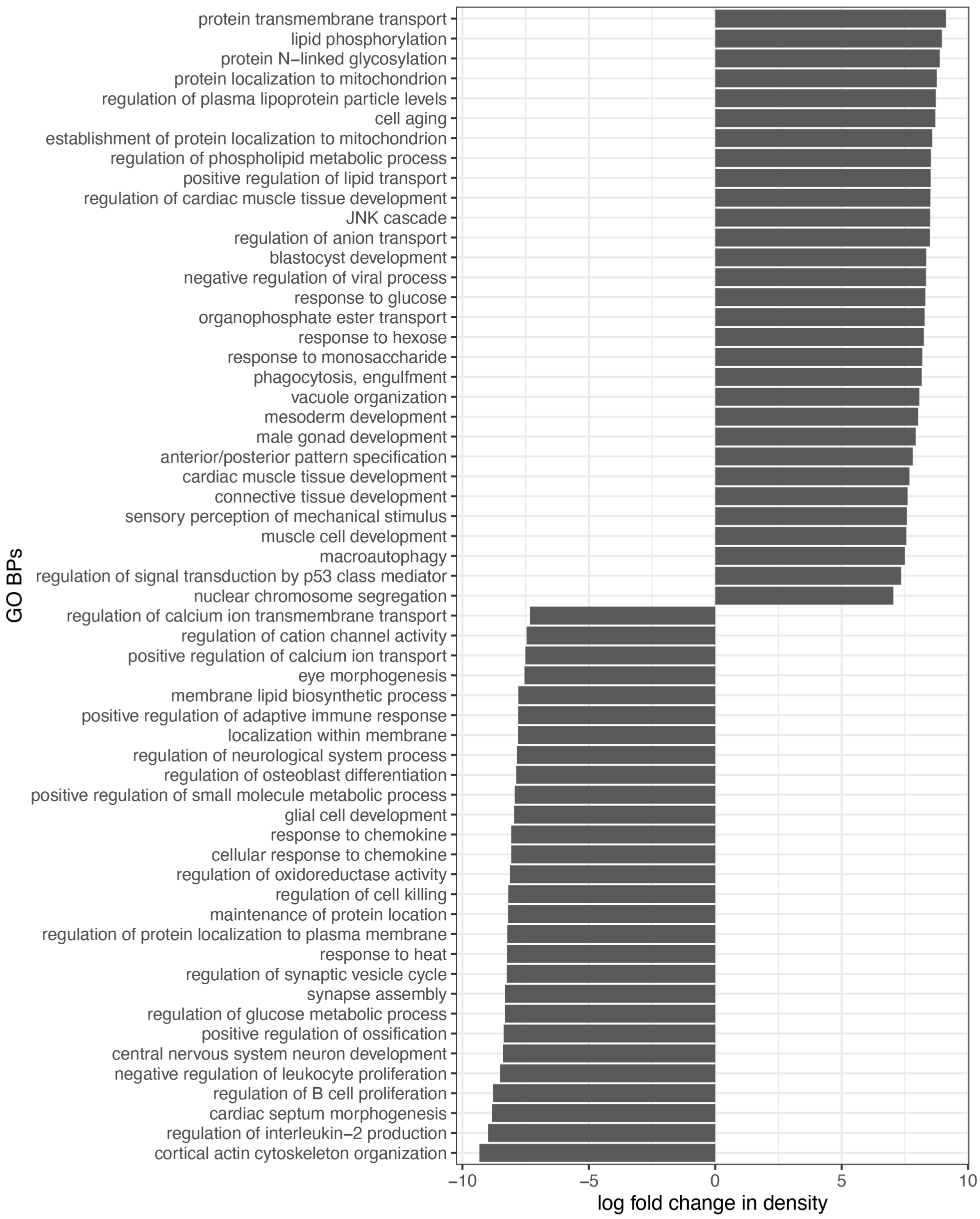

Fig C. Barplot showing GO BP terms that gain or loss cohesiveness measured as change in network density between control and AD networks of microglia.

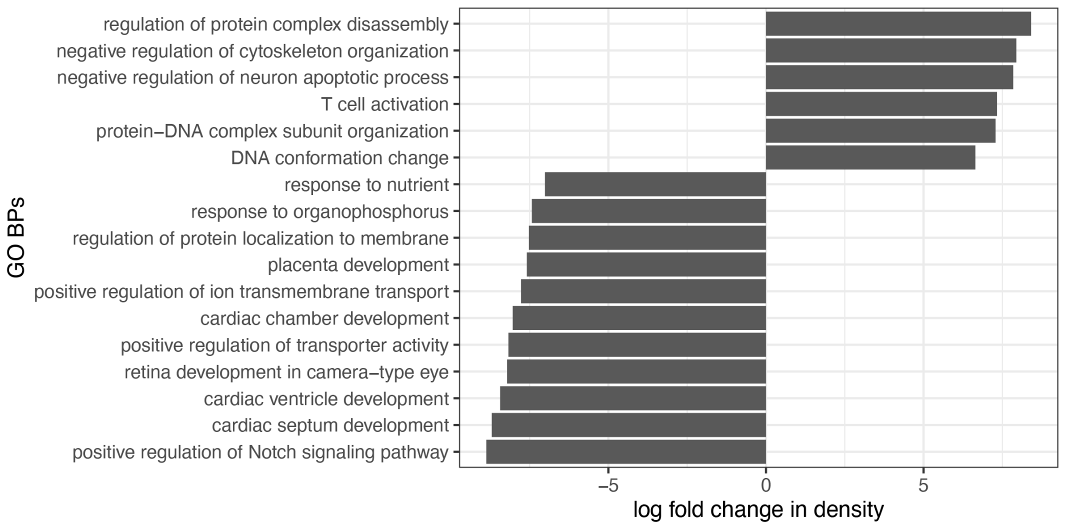

Fig D. Barplot showing GO BP terms that gain or loss cohesiveness measured as change in network density between control and AD networks of oligodendrocytes.

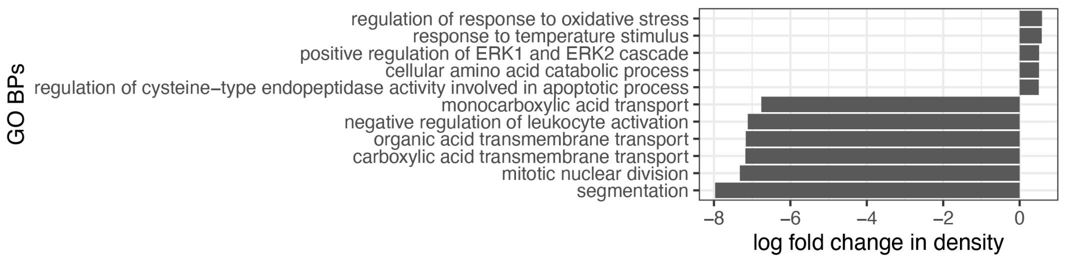

Fig E. Barplot showing GO BP terms that gain or loss cohesiveness measured as change in network density between control and AD networks of inhibitory neurons

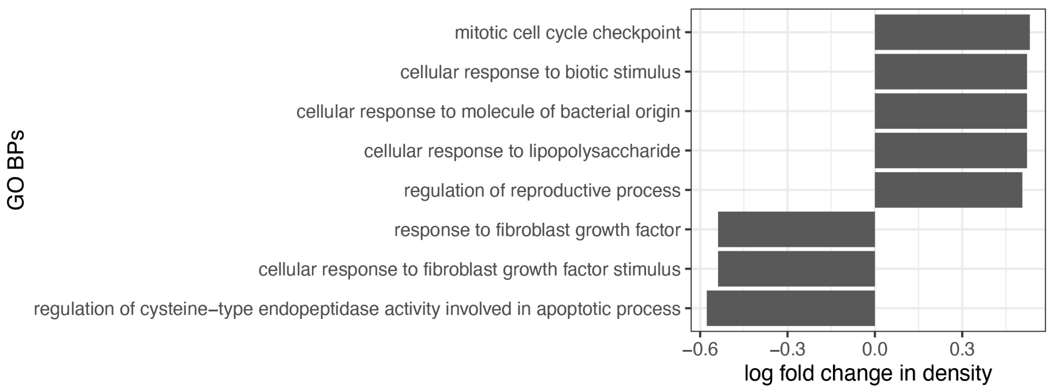

Fig F. Barplot showing GO BP terms that gain or loss cohesiveness measured as change in network density between control and AD networks of excitatory neurons.

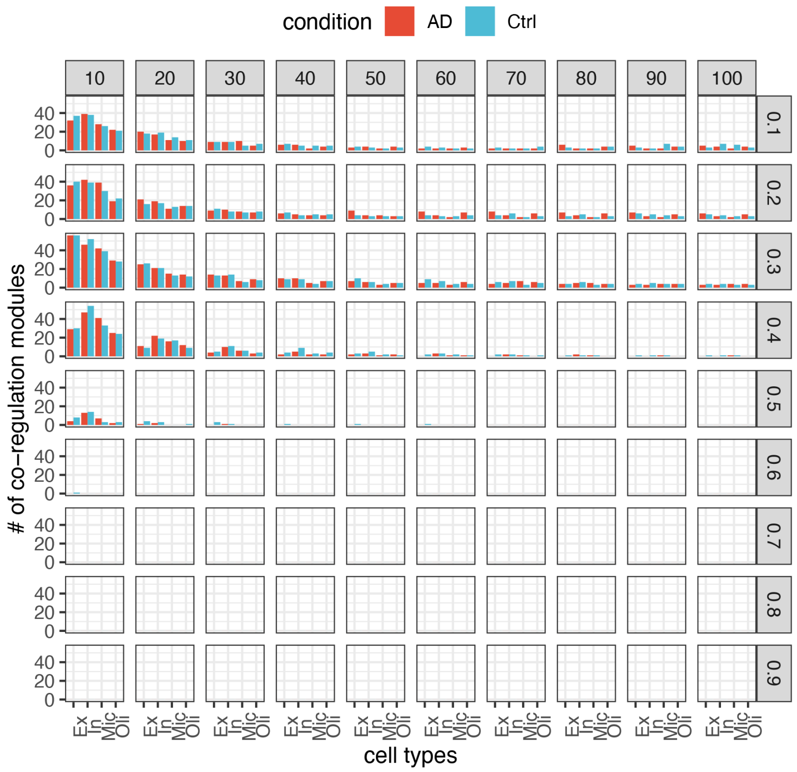

Fig G. Barplots showing the number of co-regulated gene modules (left y-axis) detected at various levels of edge-weight threshold (right y-axis) and minimum module size cut-off (top x-axis) across cell types (x-axis)

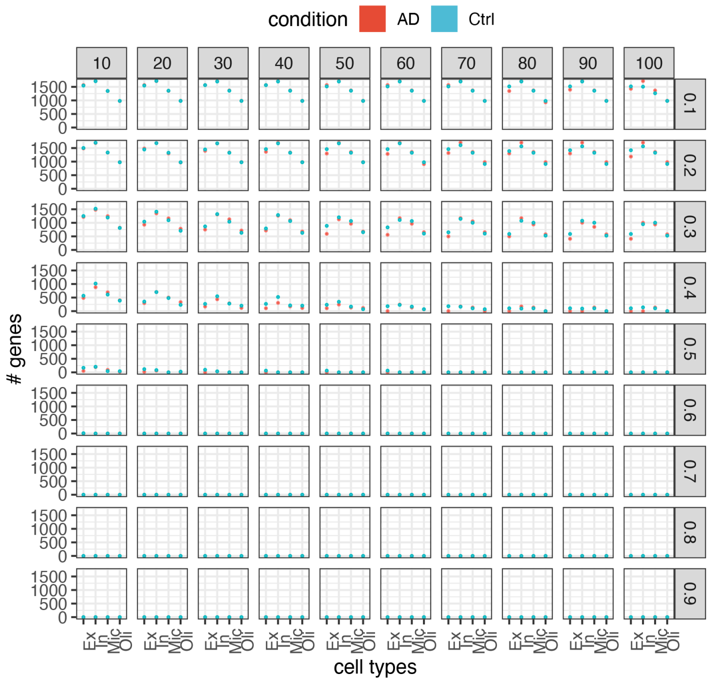

Fig H. Barplots showing the number of genes included in modules (left y-axis) detected at various levels of edge-weight threshold (right y-axis) and minimum module size cut-off (top x-axis) across cell types (x-axis)

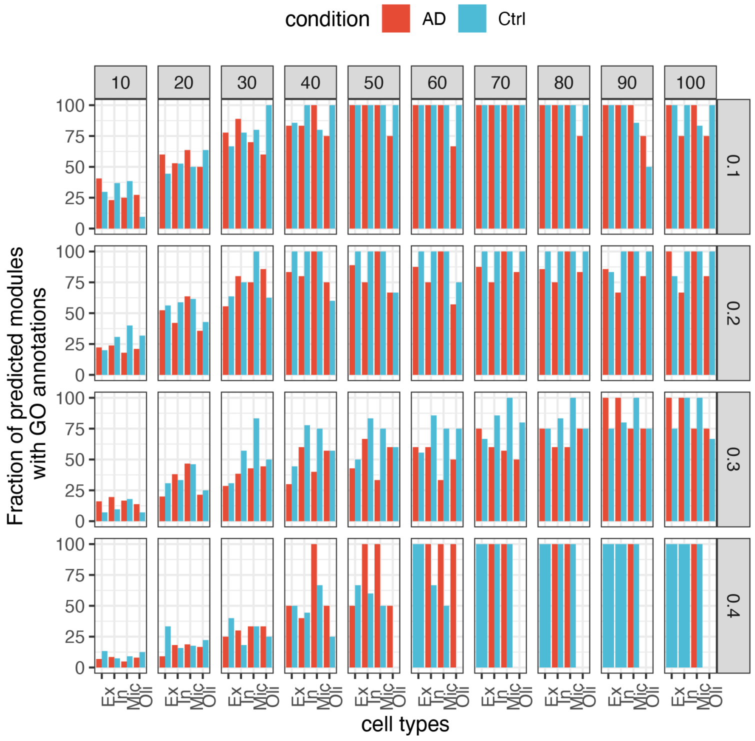

Fig I. Barplots showing the fraction of functionally annotated modules (left y-axis) detected at various levels of edge-weight threshold (right y-axis) and minimum module size cut-off (top x-axis) across cell types (x-axis).

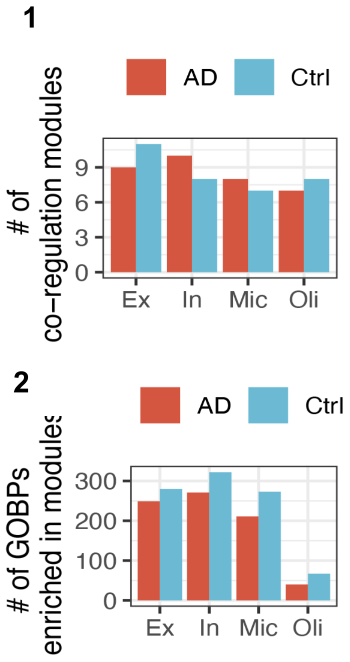

Fig J. Barplots showing (**1**) the number of modules detected in cell type AD and control networks, and (**2**) the number of enriched GO BP within those modules.

1

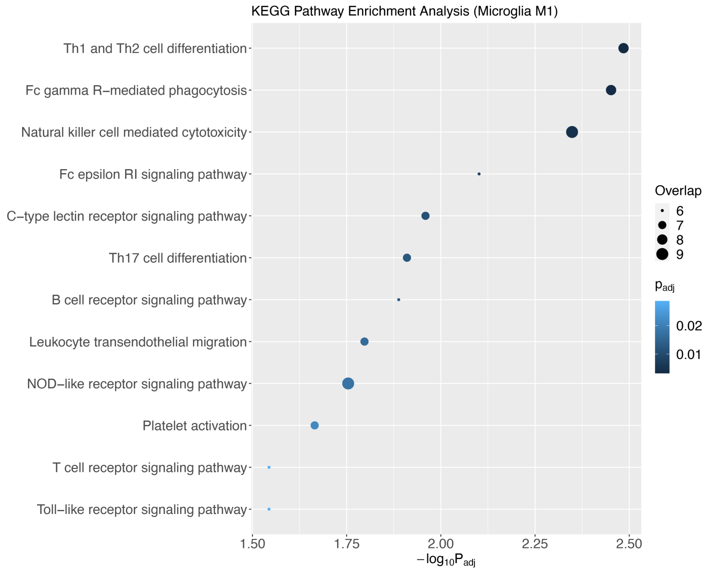

2

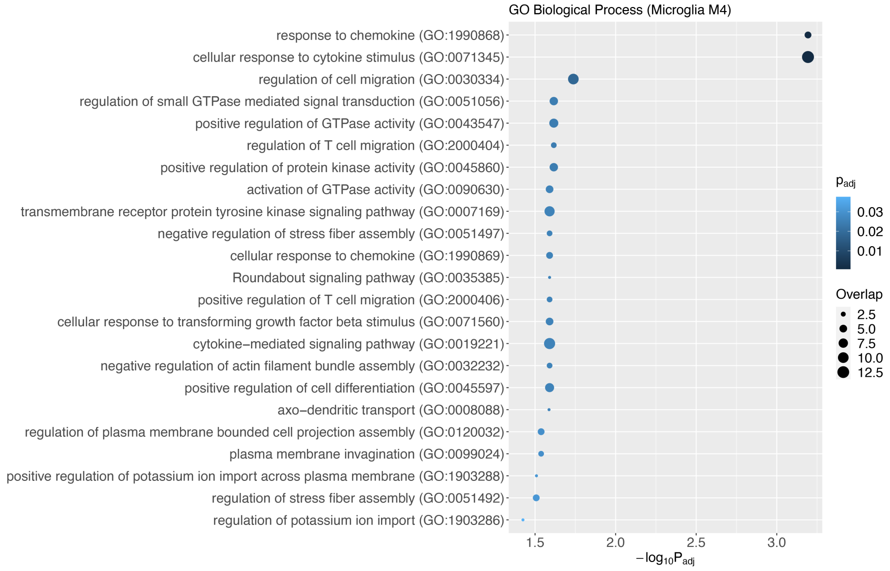

Fig K. (**1**) KEGG pathway enrichment of module M1 and (**2**) GO BP enrichment analysis of module M4 in microglia. Pathways/processes are shown on the y-axis and the adjusted p-value of enrichment is shown along the x-axis.

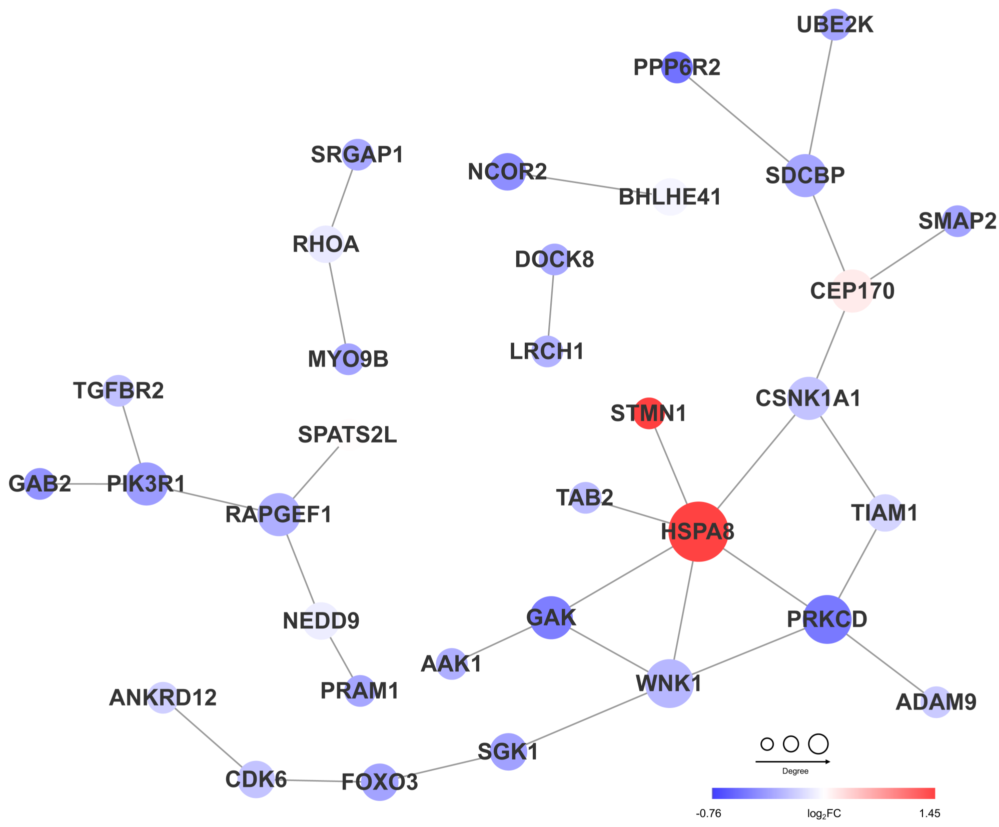

Fig L. Genes in module M4 of microglia AD network.

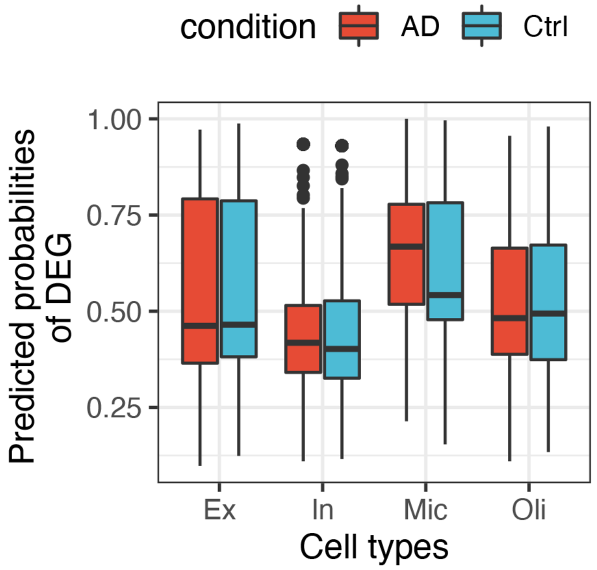

Fig M. Predicted probabilities of differentially expressed genes (DEG) being associated with AD based on the random forest-based classifier.

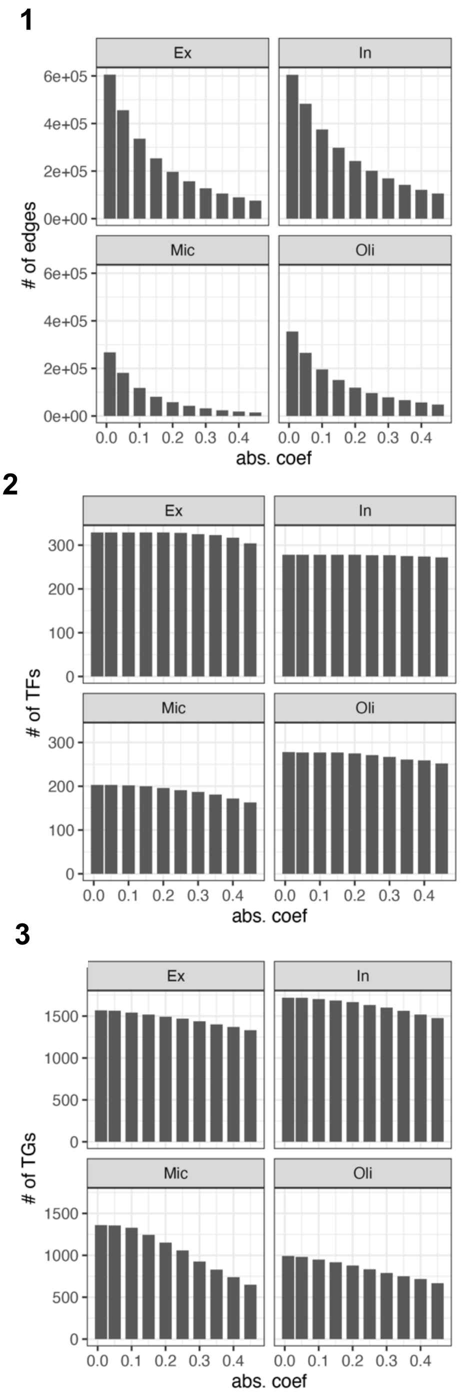

Fig N. Distribution of (**1**) edges, (**2**) TFs and (**3**) TGs within various threshold of absolute coefficients from the elastic net regression
