# Supplementary material for: Single-cell network biology characterizes cell type gene regulation for drug repurposing and phenotype prediction in Alzheimer’s disease
Source: PLoS Comput Biol. 2022 Jul 18;18(7):e1010287. doi: 10.1371/journal.pcbi.1010287 (PMC9333448; doi:10.1371/journal.pcbi.1010287)
Supplement: Attachment — Submitted filename: scNET_response_05022022.pdf [file pcbi.1010287.s011.pdf]

We have addressed the reviewer comments and provided the point-by-point responses as follows. We really appreciate the work of reviewers to help improve our manuscript.

Reviewer 1

-- Ref 1.0 Summary --

Reviewer Comment	The study by Gupta and colleagues carried out multiple levels of advanced network analyses to characterize cell-type level gene regulatory networks in Alzheimer's brains and controls and to identify the changes. The authors also developed new machine learning models to classify and prioritize Alzheimer's risk genes, including finding new candidates, and studied how the prioritized genes help predict clinical phenotypes. They further applied their recent network drug repurposing approaches to the regulatory networks to identify potential drugs. The study is highly original, comprehensive, novel, and represents significant computational advance. The network methods are very interesting to computational investigators and the findings add significant values for understanding the gene regulatory network changes and gene-phenotype relationship in Alzheimer's brains. The manuscript is written very well and easy to follow. It is relatively long because it covers several areas. Below are some technical concerns and suggestions for improving the work.
Author Response	We thank the reviewer for a great summary. We appreciate that the reviewer acknowledges our comprehensive network analysis strategy represents a significant computational advance. We carefully considered the reviewer's comments and suggestions for improving the work as below.

-- Ref 1.1 Biological relationship of the source data --

Reviewer Comment	The biological relationship of the source data needs to be described.<br>1) The scRNA-seq data for controls and Alzheimer's diseases contained additional cell types not used in this study. Why were they excluded?<br>2) Are the Hi-C data (in ref #32) for the same cell types defined as in the snRNA-seq data?<br>3) More generally, how cell types from one data source compared to the cell types in another?<br>4) Will that "mismatch" have any potential influence on the results?<br>5) Ref #85 also has Hi-C data, any reason Hi-C data from one study is better than the other?
Author Response	We thank the reviewer for pointing out that the source data needs a better description. We explained the source data and made the following changes to the revised manuscript in response to the reviewer's comments.

<table>
<tr>
<td></td>
<td>
1) We first zeroed in on the snRNA-seq dataset by Mathys et al. (2019) which contains six major cell types (Mic, Oli, OPC, Ex, In, ad Ast). We found open chromatin regions (ATAC-seq; Corces et al) for these six cell types, but the data source from Nott et al. did not report chromatin interactions for Ast and OPC. By including these two cell types in our analysis, it would be difficult to tell if any observed changes between GRNs arise due to biological variation or technical variation. Therefore, we chose to use the four cell types that have all three data modalities available to use.
2) Apart from differences in Ast and OPC as described above, the other difference between PLAC-seq data (Nott et al.) and snRNA-seq data (Mathys et al.) is in neuronal cell types; there are separate snRNA-seq data for inhibitory and excitatory neurons, but chromatin interaction data is combined for both neuronal types (and sub-types). Therefore, the differences between the inhibitory and excitatory GRNs in our study stem mainly from differences in gene expression rather than E-P interactions. We explicitly stated in the revised manuscript as below.
3) Comparing six cell types (Astro, Oligo, OPC, Mic, In, and Ex) from three data sources (snRNA-seq, chromatin interactions, and ATAC-seq), we found four cell types (Mic, Oli, In and Ex) common across all sources.
4) The only mismatch within the data sources is at chromatin interaction level for neuronal cell types because the data for all neuronal types were combined by the original authors of the dataset. Therefore, the observed variation between excitatory and inhibitory neuron GRNs are purely based on differences in gene expression patterns, unlike other cell types for which changes could also be attributed to distinct E-P interactions. We explicitly stated this in the revised version of the manuscript as below.
5) We chose to use chromatin interaction data from Nott et al. for two main reasons. First, interactomes in the data from Nott et al. (Table S5) are very well annotated for each cell type, whereas data from Corces et al. (Supplementary Data 9) lacks this information and we wanted to avoid additional processing steps to annotate them correctly. Our second reason was that we were not sure if reported loop calls from Corces et al. are complete or filtered for those which span an SNP within their ATAC-seq peaks. We wanted to make our reference networks (TF-TG links before the coexpression step) to be inclusive of all forms of evidence to generate dense networks.
</td>
</tr>
<tr>
<td>Excerpt From Revised Manuscript</td>
<td>Page 6: lines 26-30
However, it is important to note that the chromatin interaction data source we used does not resolve between the neuronal cell types. Therefore, the observed variation between excitatory and inhibitory neuron GRNs are purely based on differences in gene expression</td>
</tr>
</table>

	patterns, unlike other cell types for which changes could also be attributed to distinct E-P interactions.<br><br>Page 24: lines 27-34<br>In addition, we also obtained other omics data, including cell type chromatin interaction maps (Table S5[32]), transcription factor binding sites[34], and cell type open chromatin regions (Supplementary Data 9[33]). Note that two of these data sources (snRNA-seq and ATAC-seq) contain data for six cell types. However, the chromatin interaction data was available for only four cell types (combined for all neuronal types). Therefore, we chose to predict cell type GRNs for only the four major cell types for which all three data modalities could be obtained, as it would otherwise be difficult to tell if any observed changes between GRNs arise due to biological variation or technical variation.

-- Ref 1.2 Stability and robustness of the results --

Reviewer Comment	The stability and robustness of the results need to be addressed. In the study, snRNA-seq data from all 24 patients and 24 controls were combined, but there must be some variations among the brains.<br><br>1) Can the authors check how their networks vary when a subset of the samples is used?<br>2) In related, maybe the authors can perform an analysis with 12 patients x 12 patients, to show what null result of "changing" gene regulatory networks looks like.
Author Response	We thank the reviewer for this suggestion and agree that the stability and robustness of this study should be tested. To do this,<br><br>1) We compared the results of our centrality analysis on two independent datasets (ref #2 and ref #35) and found the central TFs to be significantly overlapping (P-value <= 0.001; Fig S1C). Because neuronal scRNA-seq data in ref #35 is split between multiple subtypes (Ex1 to Ex9), we could only compare two cell types (microglia and oligodendrocytes) for control datasets. We added the text to elaborate this point in the revised manuscript as below.<br>2) Additionally, we also created a random split of 50% samples in the snRNA-seq dataset we used (Mathys et al.) and found high correlations in the gene centrality scores (Fig. S1D), indicating that hubs are likely to be stable across cohorts.<br><br>We added new panels to Figure S1 and text information to the revised manuscript as below.

Excerpt From Revised Manuscript

Page 7: lines 1-23

To check if our centrality analysis is reproducible in an independent dataset, we obtained data from an earlier study that analyzed gene expression in healthy brain cell types[35]. We applied our GRN inference pipeline and centrality analysis to microglia and oligodendrocytes in this secondary dataset and found that the top central genes (top 20% across all centralities) show a high and statistically significant overlaps (permutation-test *P*--value < 0.001) with the main dataset (Fig. S1C). Because the secondary dataset has samples only from healthy brain cells, we could only compare the overlap with our control networks. Nevertheless, the high overlaps indicated that the top central genes we identified are indeed independent of the dataset specificity. Furthermore, the correlation in centrality scores of TFs is high between the full dataset and a reduced dataset comprising of only 50% of the samples from the original snRNA-seq dataset (Fig. S1D).

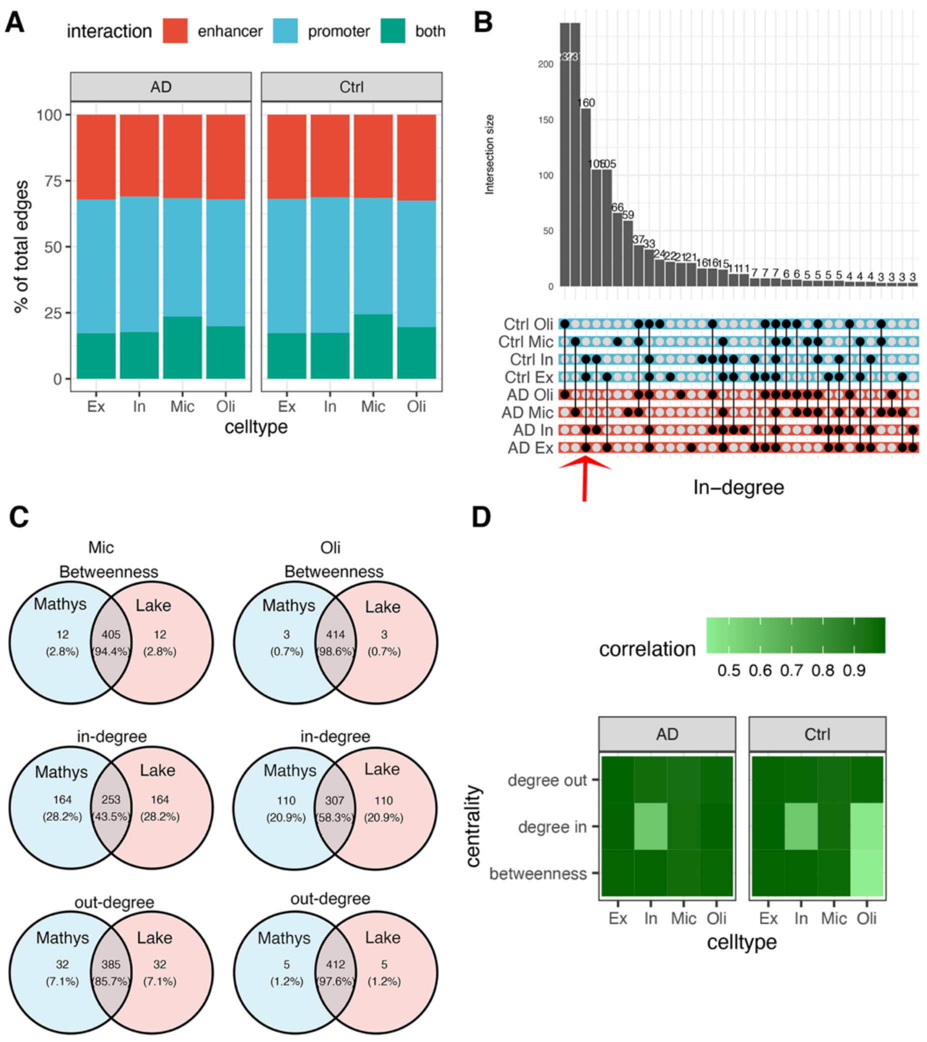

Figure S1. A) Distribution of different edge types within cell type GRNs. B) An upset plot showing overlaps between the top 10% genes

	with largest in-degree. The filled dots in the centre matrix indicate the comparison between the respective sets (along the x-axis), and the bars on the top show size of the intersection. Blue and red rows indicate control and AD, respectively. The red arrow at the bottom shows overlaps between both neuronal types across the two phenotypes. C) Overlaps between central genes in two independent datasets (Mathys et al. and Lake et al.). D) Heatmaps depicting correlation in gene centrality scores between the full dataset and a reduced dataset consisting 50% of the original samples in the snRNA-seq dataset

-- Ref 1.3 The choice of parameters --

Reviewer Comment	The choice of parameters or thresholds needs to be clarified. For example, in the scGRN analysis, “we filtered the target genes with mean square error > 0.1 and absolute elastic net coefficient < 0.01.” For people not familiar with this, it is not clear why 0.1 and 0.01 were used.
Author Response	We thank the reviewer for suggesting that a justification for the choice of thresholds should be included in the manuscript.<br><br>Given the sparse nature of single-cell datasets, we wanted to retain as many genes as possible in our networks. We expected much of the noise (i.e., indirect correlations) to be filtered because we extracted TF-TG links from three different data modalities. The main trimming of the links in our pipeline happens at the coexpression stage, for which we chose the mean squared error to be less than 0.1 to be a base filter. This choice was based on our earlier evaluation of elastic net regression to predict gene expression, which found that a subset of regulatory connections could predict the expression of most genes with a mean square error MSE of <0.05 (Wang et al. 2018, Science)<br><br>We wanted to further trim edges based on the absolute coefficient value from the elastic net regression. We tested values 0.01 to 0.45 in increments of .05 and recorded the number of TFs, TGs and edges retained at each iteration. We found that at larger values, the network loses too many genes and collapses. A sparse network would be inadequate for training ML models and score drug targets. We selected the coefficient threshold at which the first considerable drop of genes and edges occurred.<br>We added a Fig. S14 to show this and new text to the revised version of the manuscript as below.
Excerpt From Revised Manuscript	Page 25: lines 12-21<br>The *scGRNom_getNt* uses elastic net regression to infer TF-target gene edges. These unsigned edges are directed from TFs to TGs and weighted according to the strength of coexpression between the given pair of TF-TG. To identify the most optimal threshold for pruning edges, we

removed links with mean squared error (MSE) from elastic net regression > 0.1 and tested a range of absolute coefficients to further trim the edges. We found that larger values of the coefficient yield very sparse networks (very few edges and low network density), making them unsuitable for downstream analysis (Fig. S14). Therefore, we filtered the target genes with mean squared error > 0.1 and absolute coefficient < 0.01 and worked with absolute coefficient as edge weights for further analyses.

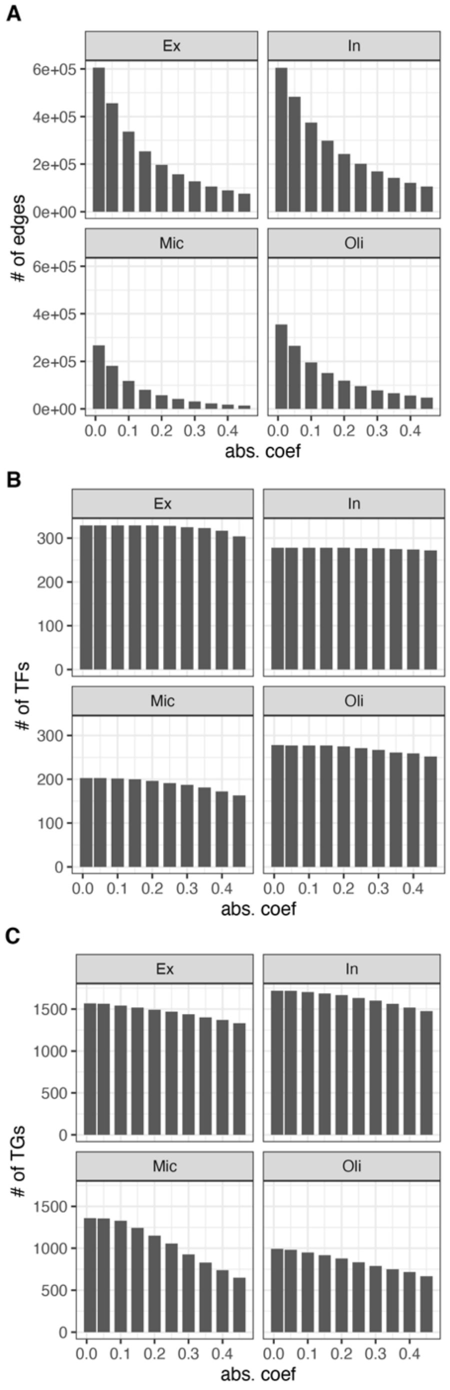

Figure S14: Distribution of A) edges, B) TFs and C) TGs within various threshold of absolute coefficients from the elastic net regression.

-- Ref 1.4 Minor comments --

Reviewer Comment	1. Do the scGRN contains both activating and inhibiting TF-target relationships?<br>2. Figure 1D is missing.<br>3. The description of Figure S1A (pg 5) seems inconsistent with what is in the figure.<br>4. Figure 2 legends for 2E and 2F need to be swapped.<br>5. Figure 3, can the authors make better visualization to support “tri-model” in 3A? The finding of so many master regulators is counterintuitive. 3D and 3E show average changes. Do they come with some variations?<br>6. In the drug repurposing analysis, do the connections of a drug to genes consider the directions of effects, i.e., activating or inhibiting genes?<br>7. In pg 21, which supplementary figures were meant to be cited in “Sup Fig.?”<br>8. n the scNET GitHub page, the authors list dependent libraries. Can they include version requirement?
Author Response	We thank the reviewer for these suggestions that will help improve the readability of the paper.<br><br>1. scGRNs link TFs to TGs if the putative DNA binding motif of the given TF is located in the open and interacting promoter or enhancer region of the TG, and if the TF has a certain degree of coexpression with the TG in a given cell type. Because these links are unsigned, we cannot determine if the TF has an inhibiting or activating effect on the TG. However, we added this good point in Discussion as potential future work.<br>2. We fixed this typo in the revised version.<br>3. Figure S1A (S1B post revision) is an upset plot to help visualize common and unique genes among multiple lists. We highlighted the bar depicting a relatively large overlap of high-indegree TFs in neuronal types than glial with a red arrow.<br>4. We fixed this typo in main figure.<br>5. We replaced this figure with a table. We see a large number of master regulators in our networks perhaps because expression data still links many indirectly connected TF-TG pairs due to cascades of gene regulation. A better distribution of TFs at the three levels of hierarchy is has been observed with subnetworks networks derived from ChIP-seq data previously. We also replaced 3D and 3E to show the distribution of promoters and fold change of genes targeted by those TFs. We revised text citing to these plots as below.<br>6. Due to the absence of signed edges in our GRNs, the drug prediction pipeline only yielded statistically significant associations without considering directions.<br>7. Thank you for pointing out the typographic error. It was a typo. We fixed it in the revised version.<br>8. We added version requirements on the project’s github page.

Excerpt From Revised Manuscript

Page 9: lines 25-28
Interestingly, neuronal TFs at all levels seem to target a relatively larger number of promoters than glial types (Fig. 3C), but the change in expression of genes targeted by microglial TFs is more prominent than other cell types (Fig. 3D).

Page 22: lines 42-44, Page 23: lines 1-4
Our network-proximity-based drug repurposing strategy predicted candidate AD drugs from microglial modules. We anticipate that these modules could potentially have pharmacologic applications for early intervention and drug research to target specific bio-mechanisms. Our strategy does not allow us to determine if the candidate drugs have inhibiting or activating effects on the target genes due to unsigned TF-TG edges. In future, integrating directionality such as activation or inhibition in gene regulation may help deeper understand dysregulation of gene expression in AD.

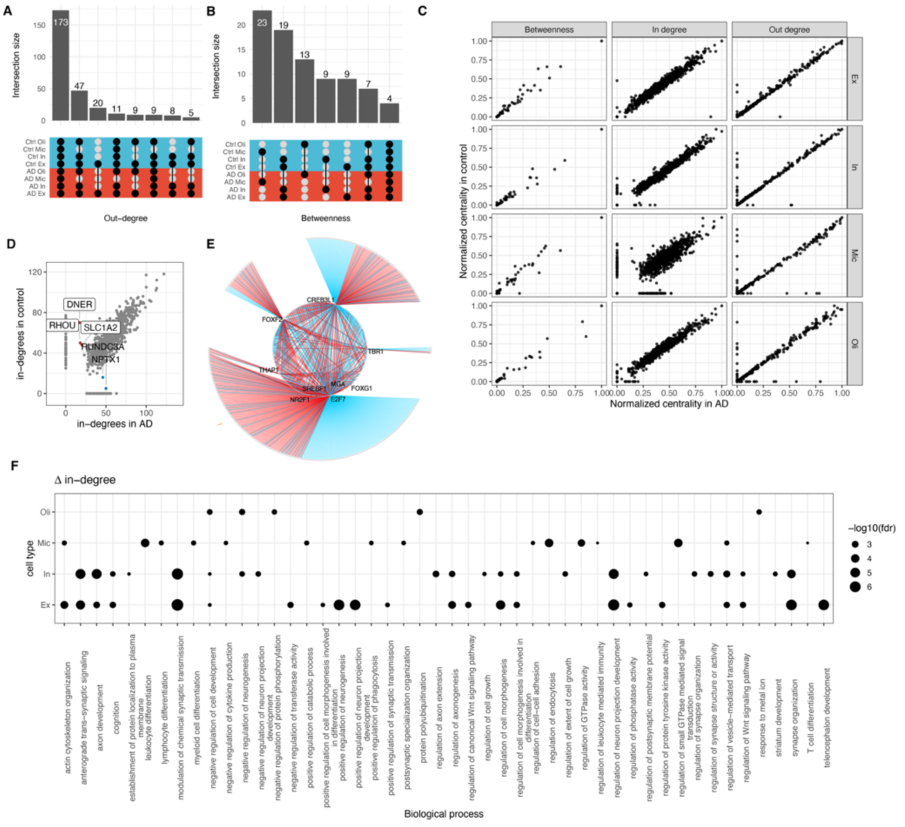

Figure 2. Gene centrality analysis. A) An upset plot showing overlaps between the top 10% genes with largest out-degree and B) betweenness centralities. The filled dots in the center matrix indicate the comparison between the respective sets (along the x-axis), and the bars on the top show size of the intersection. Blue and red rows indicate control and AD, respectively. C) Scatter plots showing normalized gene centralities distribution and D) the distribution of in-degrees in microglial AD and control networks. Genes with large changes in in-degree between AD and control are labeled. E) Visualization of the subnetwork of 9 TFs with high betweenness centrality in microglia. Grey circles around the

periphery of the network indicate target genes. Symbols of the nine central TFs are shown and the rest hidden for clarity. Blue and red edges indicate interaction in the control and AD networks, respectively. F) A dot plot showing enrichment of gene ontology biological processes (y-axis) among genes with the most extensive changes in the in-degree centrality across all cell types (x-axis). The dot size is set according to the FDR-corrected *p* values, as shown in the key.

A

Hierarchy	Top			Middle			Bottom		
Cell type/Phenotype	AD	Ctrl	Random	AD	Ctrl	Random	AD	Ctrl	Random
Ex	262	268	17	2	2	270	45	45	17
In	215	225	13	3	4	232	41	41	13
Mic	141	145	8	5	3	164	35	36	8
Oli	200	199	18	0	0	190	32	33	18

Random = 1000 trials

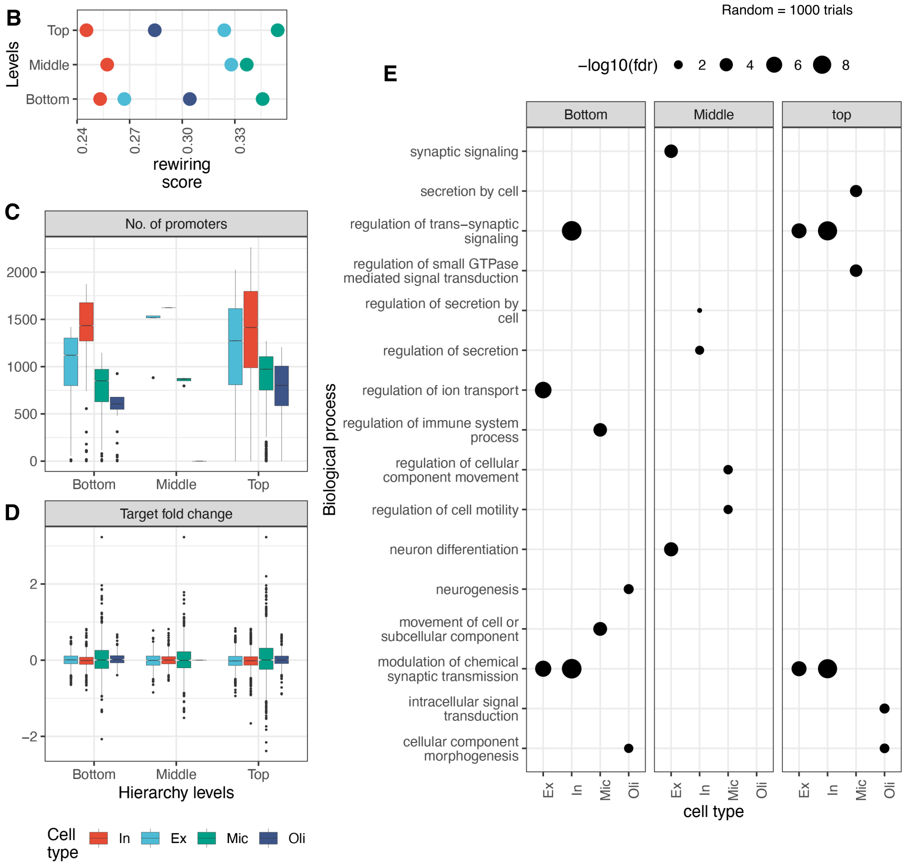

Figure 3. Hierarchy analysis of cell type GRNs. A) The distribution of TFs in three levels of hierarchy in AD, control and 1000 random GRNs across all four cell types. B) The rewiring scores of TFs (x-axis) across all three levels of hierarchies (y-axis). The distribution of C) number of promoters targeted by TFs (y-axis) and D) the fold change values (log scale; y-axis) of target genes (AD versus healthy controls) of TFs at the three levels of hierarchy (x-axes). E) Enrichment of gene ontology biological processes (y-axis) within targets of top, middle and bottom layers of the regulatory hierarchy across cell type networks (x-axis).

Reviewer 2

-- Ref 2.0 Summary --

Reviewer Comment	Authors in this manuscript present an interesting network approach to characterize cell-type gene regulation for drug repurposing and phenotype prediction in Alzheimer's disease at a single-cell resolution. While it is interesting and timely, I have the following major concerns.
Author Response	We thank the reviewer for expressing interest in our single-cell network biology approach for drug repurposing and phenotype prediction in AD. We have attempted to address the reviewer's concerns as below.

-- Ref 2.1 Central idea of this manuscript --

Reviewer Comment	I am very confused about the central idea of this manuscript.<br><br>1) Is this a new computational methodology or a biological analysis paper? There is no benchmarking and software releasing if it is a new computational method.<br>2) If this is an analysis paper, then there is no validation of the results and<br>3) reasoning of methodology selections.
Author Response	We thank the reviewer for pointing out that the central theme of the paper should stand out more.<br><br>1) This is an analysis paper. We intended to compare brain cell type gene regulatory networks (GRNs) and examine regulatory changes that occur in AD. We had three main objectives: 1) to predict cell type GRNs by integrating single-cell epigenomic and transcriptomic datasets, 2) to analyze topological and structural features of the networks and develop a disease-module-based drug repurposing strategy, and 3) to rank and prioritize genes according to their potential association with AD (Figure 1). To the best of our knowledge, this is the first study to comprehensively analyze brain GRNs across cell types and between conditions and apply it to predict drugs and prioritize genes.<br><br>2) Our gene prioritization strategy is computationally validated using independent data from the ROSMAP study. We showed that the top 5% of genes within our ranking in the microglia network (most accurate in terms of model accuracy among all cell types) can predict clinical AD phenotypes in ROSMAP with >62% accuracy, on average of multiple five-fold cross-validation runs. We believe

	this is a respectable accuracy considering we made predictions on AD genes using data from a single cell type of the human brain, essentially ignoring contributions from other cell types. Also, our results provide a resource on cell-type regulatory networks for AD, allowing the community to further design experimental validations.<br><br>3) We mostly used published tools and well-accepted network biology concepts for our analysis. For example, we applied our previously tested and published multi-omics single-cell GRN prediction pipeline to the source datasets. Other available methods for GRN inference from single cell data (e.g., SCENIC) do not consider chromatin interactions to link TFs to target genes (TGs). Network centralities, motifs, logics, and modules are standard network biology tools to estimate global and local structural and topological features of a network. Furthermore, because our networks are essentially mixed bipartite graphs, we devised a coregulation-based module discovery strategy that leverages inferred TF-TG links rather than direct coexpression patterns to detect cell type modules. We made sure that the module groupings are not random and biologically meaningful by testing multiple parameters (Fig. S7,8,9,10). We have made all code developed for this project open-source.<br><br>We made the following changes to the revised manuscript to reflect our idea better.
Excerpt From Revised Manuscript	Page 4: lines 14-28<br>In this study, we analyzed and compared the GRN characteristics of the human brain cell types and examined regulatory changes that occur in AD (Fig. 1). We integrated available single-nucleus gene expression (snRNA-seq), single-cell chromatin interaction, and open-chromatin (ATAC-seq) data to predict cell type GRNs. Specifically, we linked TFs to TGs if the putative DNA binding motif of a given TF is located in the open and interacting promoter or enhancer region of the TG, and if the TF has a certain degree of coexpression with the TG in a given cell type. Thus, TFs are linked to TGs via enhancers and promoters in two neuronal and two glial cell types in control and AD conditions (Fig. 1A). Then, we compared topological features such as gene centrality, structural features such as network motifs and logic gates, and the modular organization of each cell type GRN. We adopted a network-proximity strategy to demonstrate the utility of network modules for identification of likely drug candidates for AD (Fig. 1B). Finally, we utilized known AD genes from the published literature in a machine-learning strategy to generate a ranking of genes according to their potential association with AD. We validated these rankings on an independent bulk RNA-seq dataset (ROSMAP) (Fig. 1C).<br><br>Page 21: lines 23-32

	We applied a single-cell network biology approach to compare brain cell type GRNs and examine regulatory changes that occur in AD. We obtained multi-omics data from published resources and linked TFs to TGs if the putative DNA binding motif of a given TF is located in the open and interacting promoter or enhancer region of the TG (Hi-C loops plus ATAC-seq data), and if the TF has a certain degree of coexpression with the TG in a given cell type (snRNA-seq data). We identified cell-type-specific changes in network characteristics, such as hub genes, TF hierarchies, motifs, regulatory logics, and coregulated gene modules. Further, we revealed that using those cell type networks also improved the prediction of potential novel drug targets and AD genes, which can in turn be useful in drug repurposing and predicting clinical phenotypes of AD.<br><br>Page 23: lines 24-30<br>Our gene prioritization strategy is validated using independent data from the ROSMAP study. We showed that the top 5% of genes within our ranking in the microglia network (most accurate in terms of model accuracy among all cell types) can predict clinical AD phenotypes in ROSMAP with >62% accuracy, on average of multiple five-fold cross-validation runs. We believe this is a respectable accuracy considering we made predictions on AD genes using data from a single cell type of the brain. We anticipate a further refinement of this technique in the future.

-- Ref 2.2 Network Construction/multi-modal data --

Reviewer Comment	I have some major concerns regarding the network construction part. There is no intuition and details about how the network is constructed.<br><br>1) How different multi-omics data are used?<br>2) What are the basic summary of chromatin interactions?<br>3) How did the authors process the data (if authors downloaded it as public data, where are they and what is the pre-processing)?<br>4) Cell-type resolution of the network analysis is the paper's basis, but there is no QC of the network at all.
Author Response	We thank the reviewer for suggesting that the network construction parts be detailed.<br><br>1) The three data sources, snRNA-seq, chromatin interactions, and ATAC-seq, were used to predict cell type GRNs in the following manner. First, for each cell type, we predicted all possible interactions between enhancers and promoters for each cell type

	using the chromatin interaction data. Then, we linked TFs to TGs if the transcription factor binding sites (TFBS) is found on interacting enhancers and promoters of the TG. TFs are linked to TGs via enhancers only if the enhancer is highly accessible in the ATAC-seq data. Subsequently, we retained TF-TG links with high gene expression relationships in snRNA-seq data.<br>2) We summarised the derived edge types (enhancer, promoter, or both) in our networks. We added these bar plots as in Figure S1A in the revised manuscript.<br>3) We obtained the snRNA-seq gene count matrix from the synapse (Synapse:syn23446265). Chromatin interaction data and open-chromatin data were obtained from supplementary data files of the published articles. We added this information and details on the pre-processing steps in the revised manuscript as below.<br>4) For QC, we tested our approach on an independent dataset (Lake et al. 2018) and a random split of 50% samples from the original snRNA-seq dataset (Fig. S1C-D). For edge trimming, we tested absolute coefficient values ranging from 0.01 to 0.45 in increments of .05 and recorded the number of TFs, TGs and edges retained at each iteration. We selected the abs_coef threshold at which the first considerable drop of genes and edges occurred (information loss; Fig. S14)<br><br>In response to the reviewer's comments, we made the following changes to the manuscript.
Excerpt From Revised Manuscript	Page 5: lines 18-25<br>We predicted GRNs for four major each cell types for which all three data modalities were available. First, we predicted all possible interactions between enhancers and promoters for each cell type using the chromatin interaction data. Then, we inferred the transcription factor binding sites (TFBS) based on consensus binding site sequences in the interacting enhancers and promoters. We connected TFs to TGs via enhancers only if the enhancers are highly accessible in the ATAC-seq data. Subsequently, we retained TF-TG links that have high gene expression relationships in snRNA-seq data. Overall, we created separate control and AD GRNs for each cell type.<br><br>Page 6: lines 4-7<br>On average, ~17% of all edges in the cell type GRNs link TFs to TG via promoters, ~68% via enhancers, and ~15% via both, enhancers and promoters (Fig. S1A).<br><br>Page 7: lines 13-23<br>To check if our centrality analysis is reproducible in an independent dataset, we obtained data from an earlier study that analyzed gene expression in healthy brain cell types[35]. We applied our GRN inference pipeline and centrality analysis to microglia and oligodendrocytes in this secondary dataset and found that the top central genes (top 20% across all centralities) show a high and statistically significant overlaps (permutation-test *P*--value = 0.00001) with the main dataset (Fig. S1C). Because the secondary dataset has samples only from healthy brain

	cells, we could only compare the overlap with our control networks. Nevertheless, the high overlaps indicated that the top central genes we identified are indeed independent of the dataset specificity. Furthermore, the correlation in centrality scores of TFs is high between the full dataset and a reduced dataset comprising of only 50% of the samples from the original snRNA-seq dataset (Fig. S1D).<br><br>Page 24: lines 17-23<br>Precisely, the dataset consists of single-nucleus RNA-sequencing (snRNA-seq) of samples from the prefrontal cortex of 24 individuals diagnosed with AD pathology and 24 age-matched controls with no AD pathology. We obtained the snRNA-seq gene count matrix from synapse (Synapse: syn23446265). The original authors of the snRNA-seq dataset aggregated all 48 libraries and equalized the read depth between libraries using the CellRanger aggr pipeline before merging the data into a gene count matrix[2].<br><br>Page 25: lines 12-21<br>The *scGRNom_getNt* uses elastic net regression to infer TF-target gene edges. These unsigned edges are directed from TFs to TGs and weighted according to the strength of coexpression between the given pair of TF-TG. To identify the most optimal threshold for pruning edges, we removed links with mean squared error (MSE) from elastic net regression > 0.1 and tested a range of absolute coefficients to further trim the edges. We found that larger values of the coefficient yield very sparse networks (very few edges and low network density), making them unsuitable for downstream analysis (Fig. S14). Therefore, we filtered the target genes with mean squared error > 0.1 and absolute coefficient < 0.01 and worked with absolute coefficient as edge weights for further analysis.

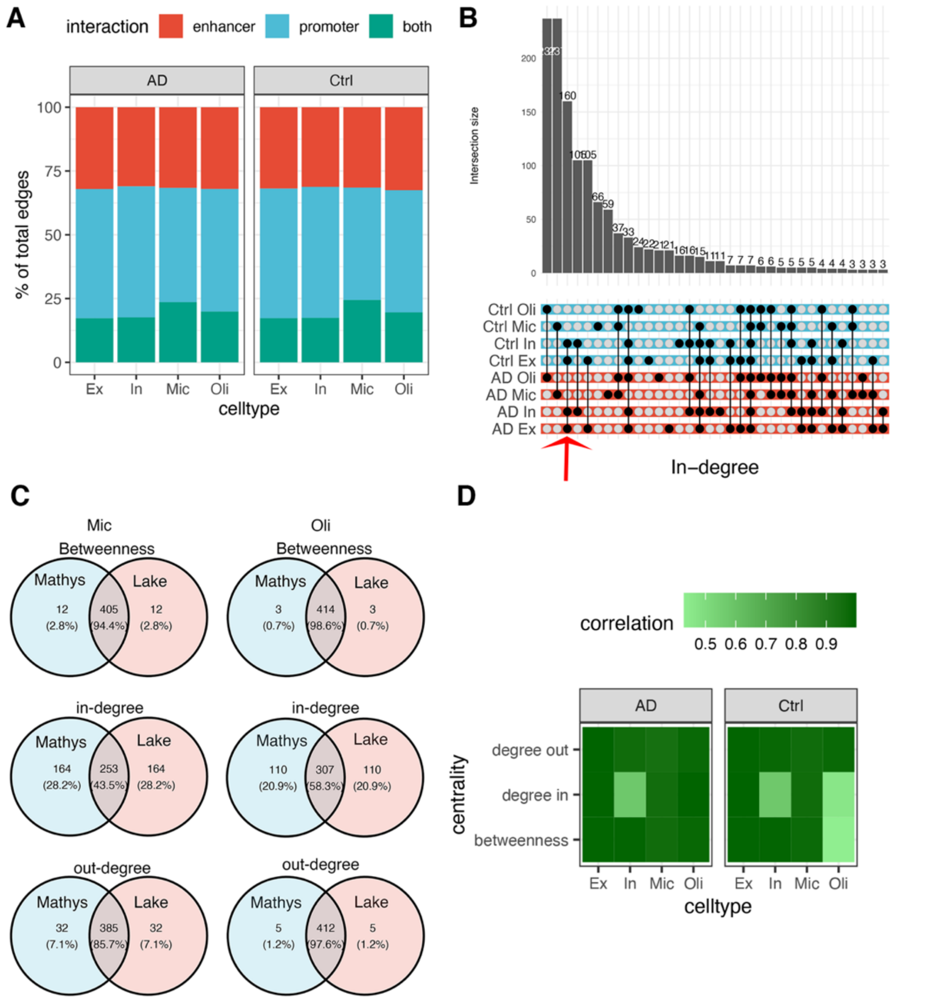

Figure S1. A) Distribution of different edge types within cell type GRNs. B) An upset plot showing overlaps between the top 10% genes with largest in-degree. The filled dots in the centre matrix indicate the comparison between the respective sets (along the x-axis), and the bars on the top show size of the intersection. Blue and red rows indicate control and AD, respectively. The red arrow at the bottom shows overlaps between both neuronal types across the two phenotypes. C) Overlaps between central genes in two independent datasets (Mathys et al. and Lake et al.). D) Heatmaps depicting correlation in gene centrality scores between the full dataset and a reduced dataset consisting 50% of the original samples in the snRNA-seq dataset.

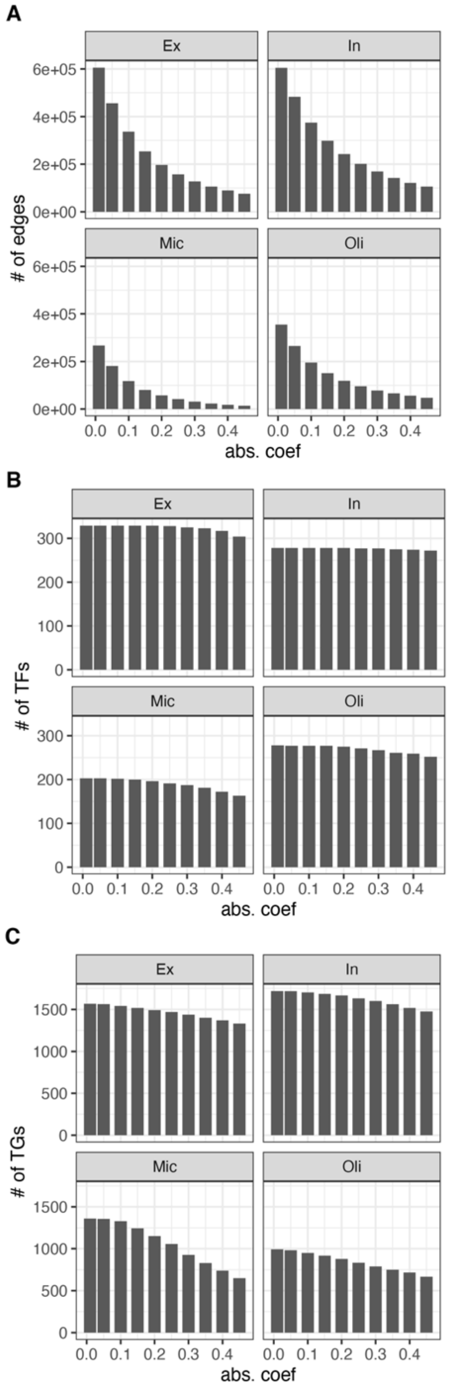

Figure S14: Distribution of A) edges, B) TFs and C) TGs within various threshold of absolute coefficients from the elastic net regression.

-- Ref 2.3 scRNA-seq data normalization --

Reviewer Comment	The authors constructed separate networks on AD and control patients. However,<br><br>1) How did the scRNA-seq data are normalized across different individuals?<br>2) There is no description of the scRNA-seq data itself. To what degree of the network differences might be due to biases?
Author Response	We thank the reviewer for suggesting that the description of the snRNA-seq normalization should be outlined.<br><br>1) We obtained the snRNA-seq gene count matrix from synapse (Synapse:syn23446265). The original authors of the snRNA-seq dataset aggregated all 48 libraries and equalized the read depth between libraries using the CellRanger aggr pipeline which normalized multiple libraries to the same sequencing depth and then recomputes the feature-barcode matrices before data merging as a gene count matrix. The exact normalization and clustering procedure is well described in the original manuscript. We downloaded the reported count matrix and worked with it to further remove lowly expressed genes and an imputation procedure to address dropouts. We normalized the count matrix using Seurat. We believe these steps are adequate to efficiently process the normalized data through the network analysis pipeline.<br><br>2) We added descriptions of the snRNA-seq data and processing steps. We also thoroughly tested the biases that may arise due to sampling size or different cohorts by testing a reduced dataset and data from an independent study, respectively (added Fig. S1 C-D).<br><br>Because these comments are similar to the previous comment (Ref. 2.2), we clubbed the changes to these two comments together.
Excerpt From Revised Manuscript	Changes shown in Ref. 2.2.

-- Ref 2.4 Biological processes in Fig. 6 --

Reviewer Comment	Some of the biological processes in Fig6 is very different to interpret as AD-related. Is there any validation of these prioritizations?
Author Response	We thank the reviewer for this comment. We realize we missed adding references to verify these observations.

	We made changes to the revised manuscript as below.
Excerpt From Revised Manuscript	Page 18: lines 39-41<br>These observations corroborate with previous findings. For instance, an increase of oxidative stress, changes in neuronal lipid metabolism, and synaptic dysfunction have been previously linked to early stage or overall AD pathology [72–74].

-- Ref 2.5 Variation in drug responses --

Reviewer Comment	Drug responses are highly personalized. How much variation is expected at the network level in AD patients?
Author Response	We thank the reviewer for suggesting a discussion on this.<br><br>We agree that drug responses are highly personalized. At the network level, the data is currently integrated across the cohort, which is just one dimension of various other variables that may play a role (e.g., genetic susceptibility, pharmacokinetic, comorbidities, etc.). At this stage, it remains challenging to predict brain-specific networks for each cell type due to limitations on sample isolation techniques resulting in data sparsity. Nevertheless, this remains a limitation of our study and should be discussed.<br><br>We added the following text to the revised manuscript as below.
Excerpt From Revised Manuscript	Page 23: lines 4-11<br>Also, it is important to note that drug responses can be highly personalized. Currently, our networks are simply abstractions of high-level multi-omics data across the cohorts, and the information contained within these networks does not resolve brain-specific rewiring. Furthermore, there are many other variables that need to be accounted for (e.g., genetic susceptibility, pharmacokinetics, comorbidities, etc.) on an individual basis through extensive clinical trials. Nevertheless, our results provide a resource on cell-type regulatory networks for AD, allowing the community to further generate hypotheses and design experimental validations.

-- Ref 2.6 linking some analysis to the goal --

Reviewer Comment	some of the analysis is difficult to be linked to the goal of this manuscript - drug repurposing and phenotype prediction in AD. For instance, how did the network motifs contribute to this goal?
Author Response	We thank the reviewer for suggesting that a better description of how results from different analysis converge to illuminate biological insights on AD brain biology.<br><br>One of our objectives was to utilize known AD genes and their network patterns to find other novel AD-associated genes. We intended to generate a ranked list of genes using a ML strategy (Fig. 6A-C) and explain the regulatory mechanisms of top-ranked genes (Fig. 6 D-H). The original version of the manuscript only connects top TFs to their hierarchy levels. We have now added information on network motifs and regulatory logics within these top TFs to further illuminate cooperativity. We also performed module enrichment of top ranked genes which further highlighted the significance of module 4 (disease module for which drug candidates were predicted).<br><br>Accordingly, we added three new panels to Figure 6 in the revised manuscript and text as below.
Excerpt From Revised Manuscript	Page 19: lines 26-32<br>Furthermore, we found that TFs in the top of our rankings participate in a significantly larger number of feed-forward loops than expected by chance (Fig. 6F). We also observed many AND and OR gates involving these TFs (Fig. 6G), indicating that some of these TFs coordinate their activities to activate target gene expression. Furthermore, we found that genes at the top of our rankings are mostly overrepresented in module 4 (M4) in microglia. This is interesting because M4 was also identified as disease-module with a significant number of known drug targets (Fig. 5E).

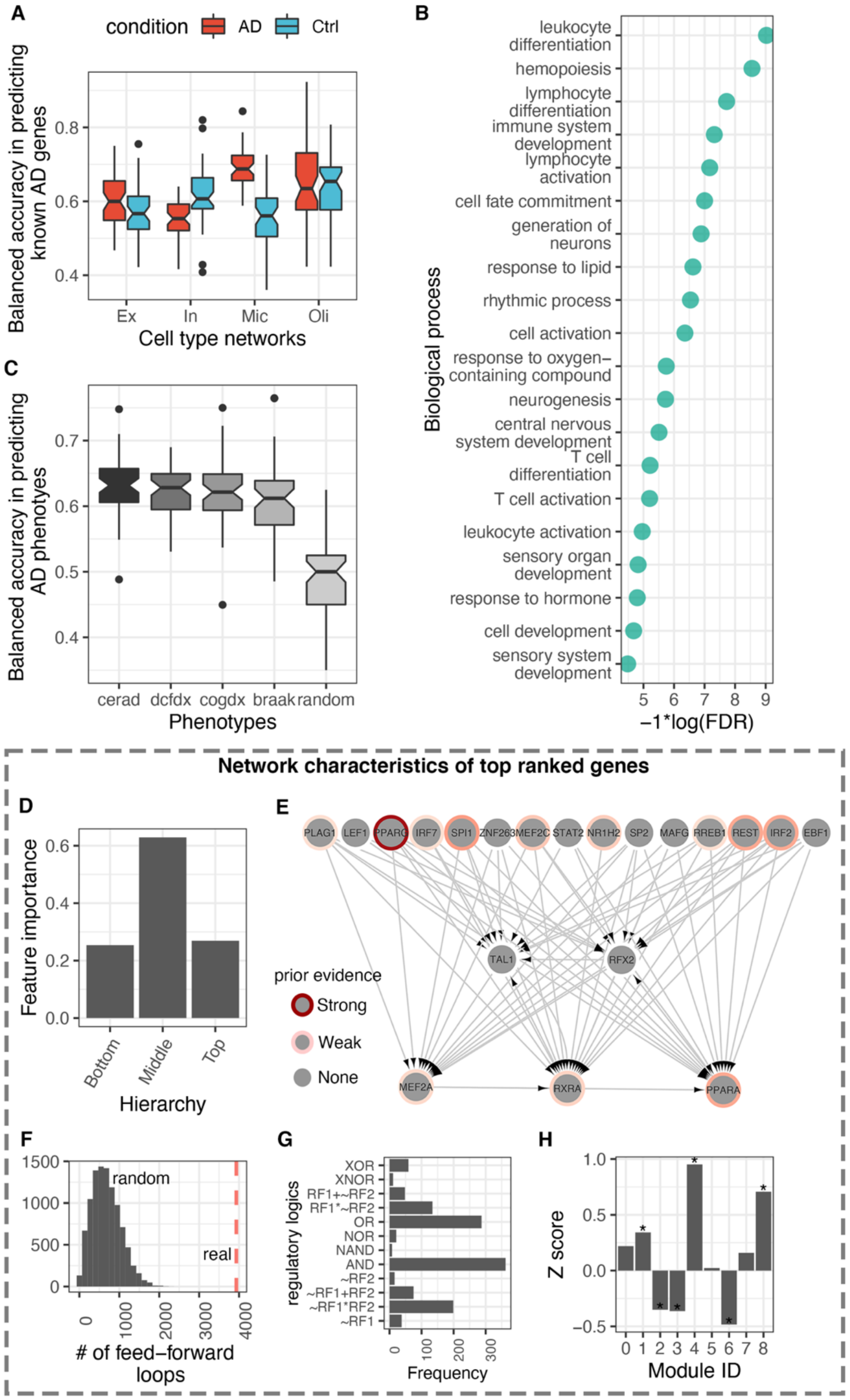

Figure 6. **Gene prioritization using supervised machine learning**. A) Boxplots showing the distribution of balanced accuracies (y-axis; obtained from 10 independent runs of five-fold cross-validation) in predicting known AD genes using interaction patterns in cell type GRNs as features (x-axis). B) Gene ontology biological process terms enriched within the top 20% predictions in the microglia AD machine learning (ML) model. The terms are depicted along the y-axis, and the FDR corrected *p*-values are shown along the x-axis. C) Genes were sorted

	according to their probability of being associated with AD in the microglia ML model, and the top 5% of the sorted list was used as features to predict AD phenotypes in an independent dataset (ROSMAP). The boxplots show the distribution of balanced accuracies (y-axis) obtained from testing four AD phenotypes (see Methods) and a set of randomly selected samples (x-axis). D) Average feature importance scores of TFs at the three hierarchy levels in the microglia AD network. E) Visualization of the subnetwork connecting top 10% TFs with highest feature importance scores in microglia AD network. Each grey node depicts a TF with border color set along a red gradient according to the disease-gene association score given in the DisGeneNet database (based on preliminary evidence collected from independent studies). F) Feed-forward loops observed within top-ranked TFs in the microglia ML model (red line) and the distribution in 1000 random networks (grey bars).G) Regulatory logics observed within top-ranked TFs in the microglia. H) Enrichment of top-ranked genes within coregulated genes modules in microglia (* Permutation p value < 0.001).
